# Thrombectomy with and without computed tomography perfusion imaging for large-vessel occlusion stroke in the extended time window: a meta-analysis of randomized clinical trials

**DOI:** 10.3389/fneur.2023.1185554

**Published:** 2023-08-17

**Authors:** Zheng Zhan, Feng Gu, Yi Ji, Yu Zhang, Yi Ge, Zhong Wang

**Affiliations:** ^1^Department of Neurosurgery and Brain and Nerve Research Laboratory, The First Affiliated Hospital of Soochow University, Suzhou, Jiangsu, China; ^2^Department of Neurology, The Affiliated Changzhou Second People's Hospital of Nanjing Medical University, Changzhou, Jiangsu, China

**Keywords:** large-vessel occlusion stroke, NCCT, CTP, prognosis, meta-analysis

## Abstract

**Objective:**

In recent years, several studies have used computed tomography perfusion (CTP) to assess whether mechanical thrombectomy can be performed in patients with large-vessel occlusion (LVO) stroke in an extended time window. However, it has the disadvantage of being time-consuming and expensive. This study aimed to compare the impact of the CTP group with the non-CTP group [non-contrast CT (NCCT) ± CT angiography (CTA)] on the prognosis of this patient population.

**Methods:**

A search of PubMed, EMBASE, and the Cochrane Library databases was conducted to collect randomized controlled trials (RCTs) comparing the two strategies. Outcome indicators and factors influencing prognosis were summarized by standardized mean differences, ratios, and relative risks with 95% confidence intervals using a random-effects model.

**Results:**

A total of two RCTs were included in the combined analysis. There were no significant differences in the main outcome indicators (modified Rankin Scale score at 90 days, successful postoperative reperfusion rate) or the incidence of adverse events (90-day mortality and symptomatic intracranial hemorrhage) between the NCCT ± CTA and CTP groups. The time from the last puncture appeared to be significantly shorter in the NCCT ± CTA group than in the CTP group (SMD: −0.14; 95% CI: −0.24, −0.04). Among them, age (OR: 0.96; 95% CI: 0.94, 0.98), ASPECTS (OR: 1.18; 95% CI: 1.12, 1.24), NIHSS score (OR: 0.90; 95% CI: 0.89, 0.91), and diabetes (OR: 0.69; 95% CI: 0.54, 0.88) were associated with a 90-day independent functional outcome.

**Conclusion:**

These findings suggest that the choice of NCCT ± CTA (without CTP) for the assessment of mechanical thrombectomy within 6–24 h after LVO in the anterior circulation is not significantly different from CTP; instead, the choice of NCCT ± CTA significantly reduces the time from onset to arterial puncture.

## 1. Introduction

Stroke is a widely prevalent disease that affects one-quarter of the population during their lifetime. As the second leading cause of death and the third leading cause of disability in adults worldwide, it has attracted the attention of many healthcare professionals (1). In patients with acute ischemic stroke, endovascular treatments such as mechanical thrombectomy and pharmacological thrombolysis have been shown to improve the functional outcome of stroke patients (2). Recent results from the DAWN (DWI or CTP Assessment with Clinical Mismatch in the Triage of Wake-Up and Late Presenting Strokes Undergoing Neurointervention with Trevo) (3) and DEFUSE3 (Endovascular Therapy Following Imaging Evaluation for Ischemic Stroke) (4) trials suggest that the use of perfusion imaging for endovascular treatment selection within an extended time window may be extremely beneficial for stroke patients. However, the two trials required all patients to only undergo computed tomography perfusion (CTP) or magnetic resonance imaging (MRI) of the brain. The importance of advanced perfusion imaging (CT perfusion or MRI) is reflected in the extended time window (6–24 h after stroke onset) by providing an assessment of ischemic tissue viability beyond an arbitrary clock time to allow physicians to select treatment modalities to resuscitate the patient (5). However, for low-level stroke centers, emergency MRI and CTP are not fully implemented (6). Therefore, a more pragmatic and resource-efficient approach to selecting patients is needed.

Multimodal CT imaging, which includes non-contrast CT (NCCT), CT angiography (CTA), and CT perfusion (CTP), is also crucial to the diagnosis and treatment of acute ischemic stroke (7). Previous studies have found no significant difference in the accuracy of NCCT-based Alberta Stroke Program Early CT Score (ASPECTS) and CTP in predicting lesion volume in the hyperacute phase of ischemic stroke (8). The use of ASPECTS in combination with different collateral scores, such as the National Institutes of Health Stroke Scale (NIHSS) to determine ischemic viability, provides an alternative to advanced imaging techniques within an extended time window. Previous studies have also demonstrated that CT may be more sensitive than CTP to detect irreversible damage during the extended time window (9). In addition, clinical outcomes have been observed in stroke patients selected by optimal CTP parameters, but this increased overall costs and prevented other patients from receiving care who would have benefited from it (10).

In this article, we review the different imaging modalities proposed in the literature and perform a meta-analysis of the utility and limitations of CT and CTP as endovascular treatment options. The feasibility and potential benefits of using only NCCT ± CTA in an extended time window were discussed.

## 2. Methods

This meta-analysis conforms to the Preferred Reporting Items for Systematic Reviews and Meta-Analyses (PRISMA) regulatory process (11).

### 2.1. Search strategy

Two researchers identified all articles containing the terms through PubMed, EMBASE and Cochrane Library databases, “CT,” “CTP,” and “thrombectomy” in the title or abstract. We also checked reviews and references of other studies to avoid potentially missing studies. The searched articles were all published before November 2022.

### 2.2. Study selection

The inclusion criteria for the studies included in this article are as follows: (1) Participants: the study cohort included consecutive patients who met the following criteria: baseline NIHSS score of 6 or more, occlusion of the internal carotid artery or proximal middle cerebral artery (M1/M2 segments), pre-stroke modified Rankin Scale (mRS) score of 0 to 2, and time last seen well to treatment of 6 to 24 h. (2) Intervention: Compared with the control group, the experimental group used NCCT ± CTA instead of CTP in the selection of patients for endovascular treatment. (3) Outcome: The primary outcome was the distribution of the mRS score at 90 days (ordinal shift analysis). Secondary clinical outcomes included the rate of 90-day functional independence (mRS scores of 0–2), and successful reperfusion, defined as a grade 2b or 3 (>50% of the affected territory) on the modified Treatment in Cerebral Infarction scale. A standard approach to mRS assessment was used. Safety endpoints included post-procedural symptomatic intracranial hemorrhage (as defined in the European Cooperative Acute Stroke Study III: intracranial hemorrhage that is associated with deterioration in NIHSS ≥ 4 points and the main cause for neurological deterioration) and 90-day mortality. (4) Study design: RCT comparing the efficacy and safety of selecting acute large-vessel stroke patients for endovascular treatment between the NCCT ± CTA group and the CTP group in the extended time window.

### 2.3. Data extraction and quality assessment

We collected relevant data from various studies we needed, namely the article title, name of the first author, year of publication, country, study design, baseline characteristics of included studies, and change in the mean value of each quantitative indicator from baseline to endpoint. Two investigators critically checked the data for each study. If the data from the included studies were not publicly available, we searched the ClinicalTrails.gov database for the raw data. The risk of bias was evaluated through the Cochrane Collaboration's tool, including selection bias, performance bias, detection bias, attrition bias, reporting bias, and other biases.

### 2.4. Statistical analysis

R with the “meta” package was used to analyze the data. We visualized the aggregated results for each endpoint event using a forest plot diagram. The standardized mean difference (SMD) with a 95% confidence interval (CI) was computed for the difference between baseline and endpoint in each indicator. The odds ratio (OR) with 95% CI values was calculated for adverse events. Cochrane's Q test and I^2^ were used to calculate outcome heterogeneity. We used a fixed-effects model when heterogeneity was low (I^2^ < 50%, *P*-value > 0.10); otherwise, we used a random-effects model.

## 3. Results

### 3.1. Search results

**Figure 1:**
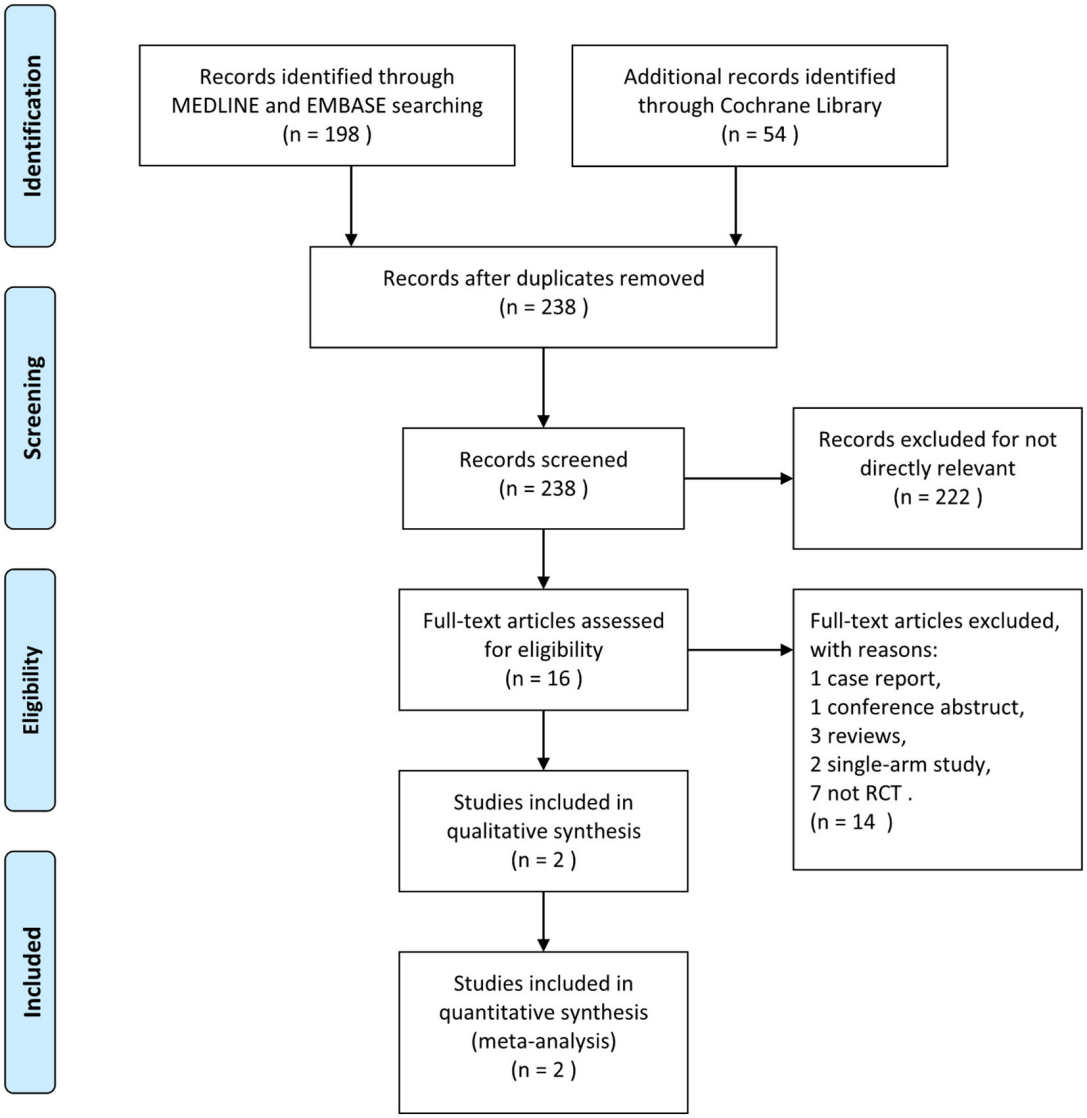
The PRISMA flow diagram.

Based on the search strategy, we retrieved 252 potentially relevant records, 14 of which were duplicates. After the screening, 222 articles were excluded for irrelevant content. Of the remaining 16 articles, only two RCTs were ultimately included in the meta-analysis based on the inclusion and exclusion criteria, and their main characteristics are shown in Table 1. The study by Nguyen et al. (12) included data from 1,286 patients in Europe and North America between 2014 and 2022, with a ratio of 1:1.4 patients undergoing NCCT ± CTA vs. those undergoing CTP. Another study included data from 247 patients in North America, Europe, and Asia between 2013 and 2017, with a ratio of 1:2.7 patients receiving NCCT ± CTA vs. CTP. The entire search flowchart is shown in Figure 1. A total of 1,533 eligible patients were included, including 602 in the NCCT ± CTA group and 931 in the CTP group. The age distribution of the two studies was 50–81 years. The proportion of women was slightly higher than that of men in both studies.

**Table 1:** Included studies and their characteristics.

**Study**	**Unique identifier**	**Years**	**Site**	**Total patients**	**NCCT ±CTA/CTP**
Nguyen et al. (12)	NCT04096248	2014–2020	Europe and North America	1,286	1:1.4
Nogueira et al. (6)	NCT02040259	2013–2017	North America, Europe, Asia	247	1:2.7

### 3.2. Quality assessment and risk of bias

The overall quality of the included studies was high. They consisted of RCTs registered on ClinicalTrials.gov. According to the risk of bias assessment, the *post-hoc* retrospective design of the two studies may lead to some selection bias, making it difficult to generalize the results. Neither study had an independent imaging assessment center, and there may be some variation in the interpretation of imaging results, selection of imaging modalities, and automated CTP processing software between providers, resulting in bias. Although both studies encouraged the consecutive enrollment of patients, they were not continuously monitored. Additionally, both studies were limited to patients with internal carotid artery occlusion or middle cerebral artery M1/M2 segment occlusion.

### 3.3. Assessment of the primary outcome

**Figure 2:**
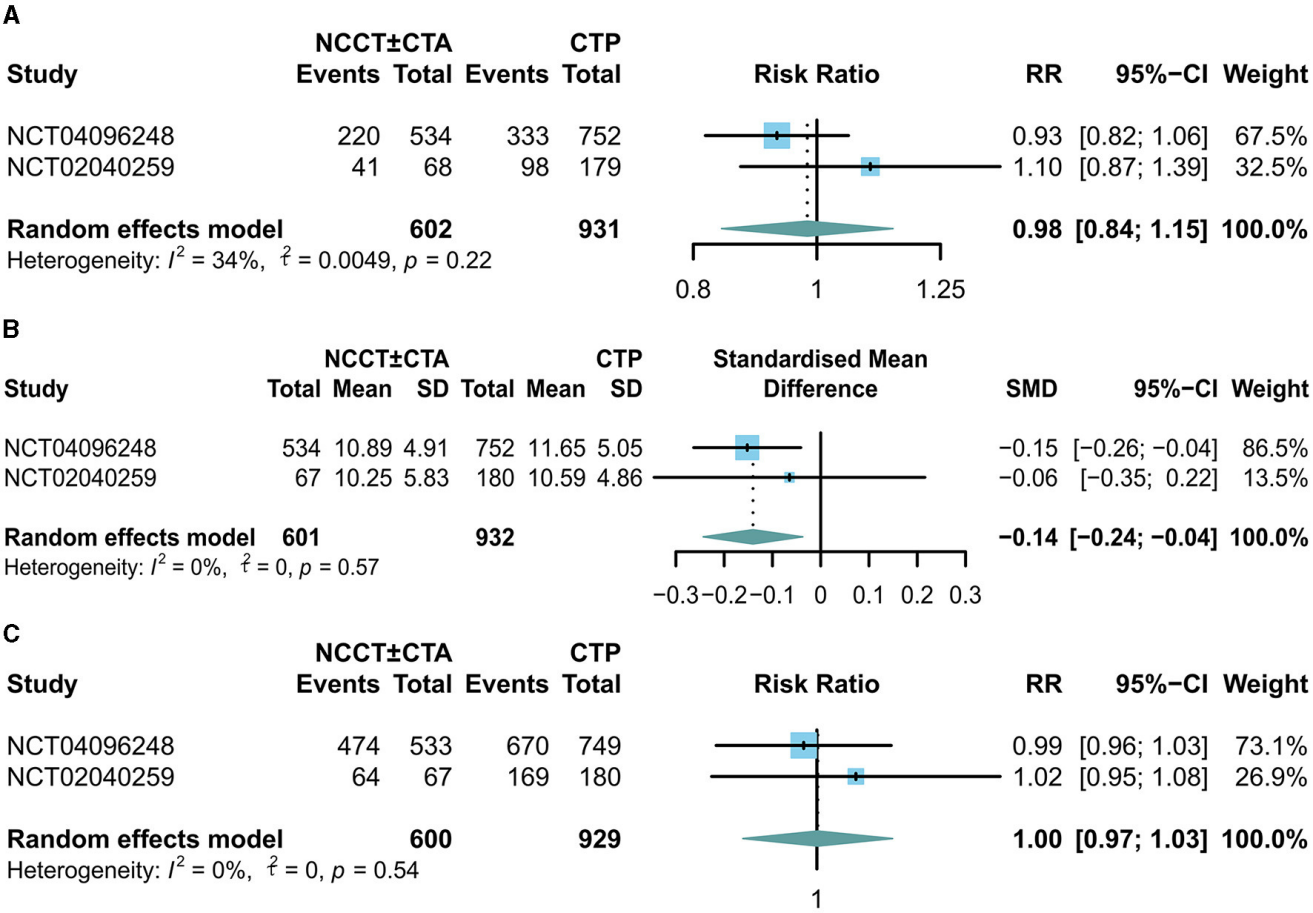
Forest plots for the odds of **(A)** good outcome (90-d mRS score 0-2), **(B)** time last seen well to puncture, and **(C)** successful postoperative reperfusion rate (mTICI ≥ 2b).

**Figure 3:**
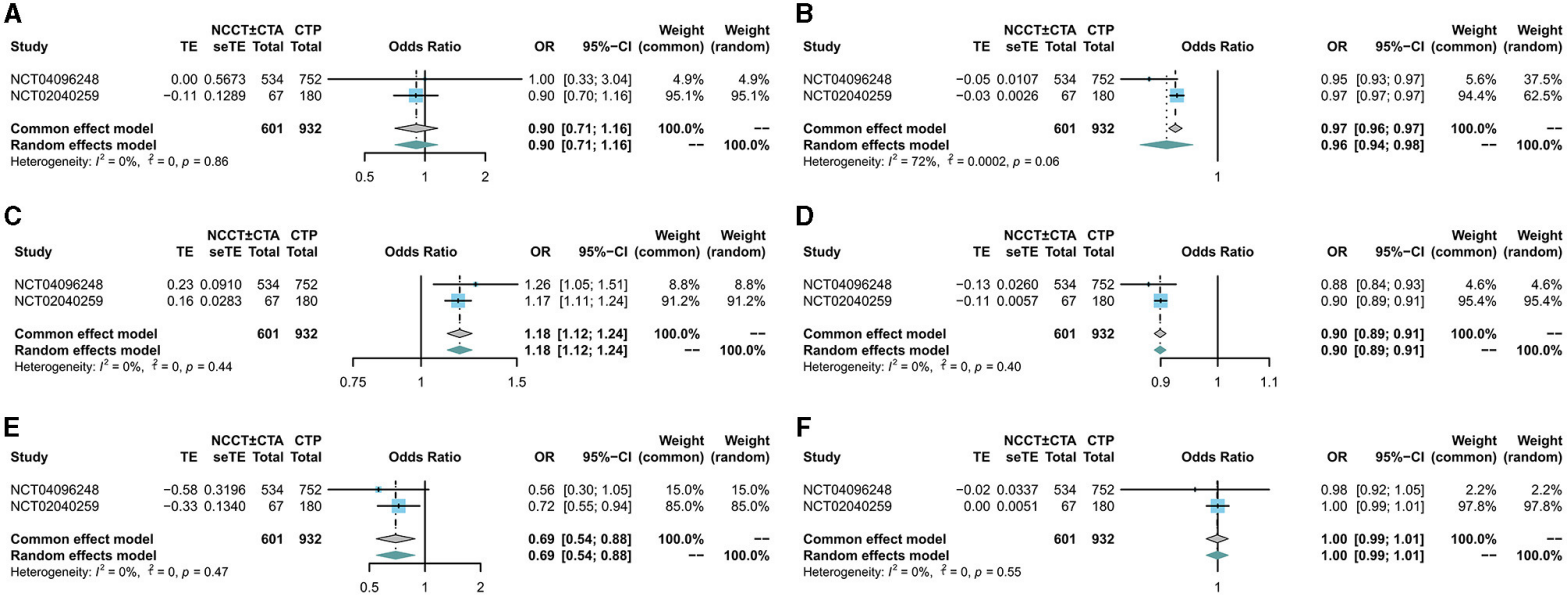
Forest plot for multivariate analysis of good prognosis. The diamond indicates the odds ratio (95% confidence interval) for all patients together. **(A)** CTP, **(B)** age, **(C)** ASPECTS score, **(D)** NIHSS score, **(E)** diabetes, **(F)** time last seen well to puncture.

There was no significant difference in mRS scores between the NCCT ± CTA and CTP groups at 90 days of follow-up (RR: 0.98; 95% CI: 0.84, 1.15) in Figure 2A. Patients in the NCCT ± CTA group had a significantly shorter time from last seemed well to puncture than those in the CTP group (SMD: −0.14; 95% CI: −0.24, −0.04; Figure 2B). However, there was no significant difference in the successful postoperative reperfusion rate (mTICI ≥ 2b) between the two groups in Figure 2C (RR: 1.00; 95% CI: 0.97, 1.03). Pooling the results of the multivariate dichotomous mRS study (good outcome defined as a 90-day modified Rankin Scale score of 0 to 2) from the two RCT studies found no significant difference in the incidence of good prognosis between patients who chose CTP as their imaging modality and those who chose NCCT ± CTA as their imaging modality (OR: 0.90; 95% CI: 0.71, 1.16; Figure 3A). In addition, lower age (OR: 0.96; 95% CI: 0.94, 0.98; Figure 3B), higher ASPECTS (OR: 1.18; 95% CI: 1.12, 1.24; Figure 3C), lower NIHSS scores (OR: 0.90; 95% CI: 0.89, 0.91; Figure 3D), and non-diabetic patients (OR: 0.69; 95% CI: 0.54, 0.88; Figure 3E) were more likely to have a 90-day independent functional outcome. Moreover, the length of time from the last time the patient looked good to the puncture (OR: 1.00; 95% CI: 0.99, 1.01; Figure 3F) was not significantly correlated with a good prognostic outcome. The results of the 90-day ordinal mRS shift analysis were similar to the above findings, as shown in the Supplementary material.

### 3.4. Adverse events

**Figure 4:**
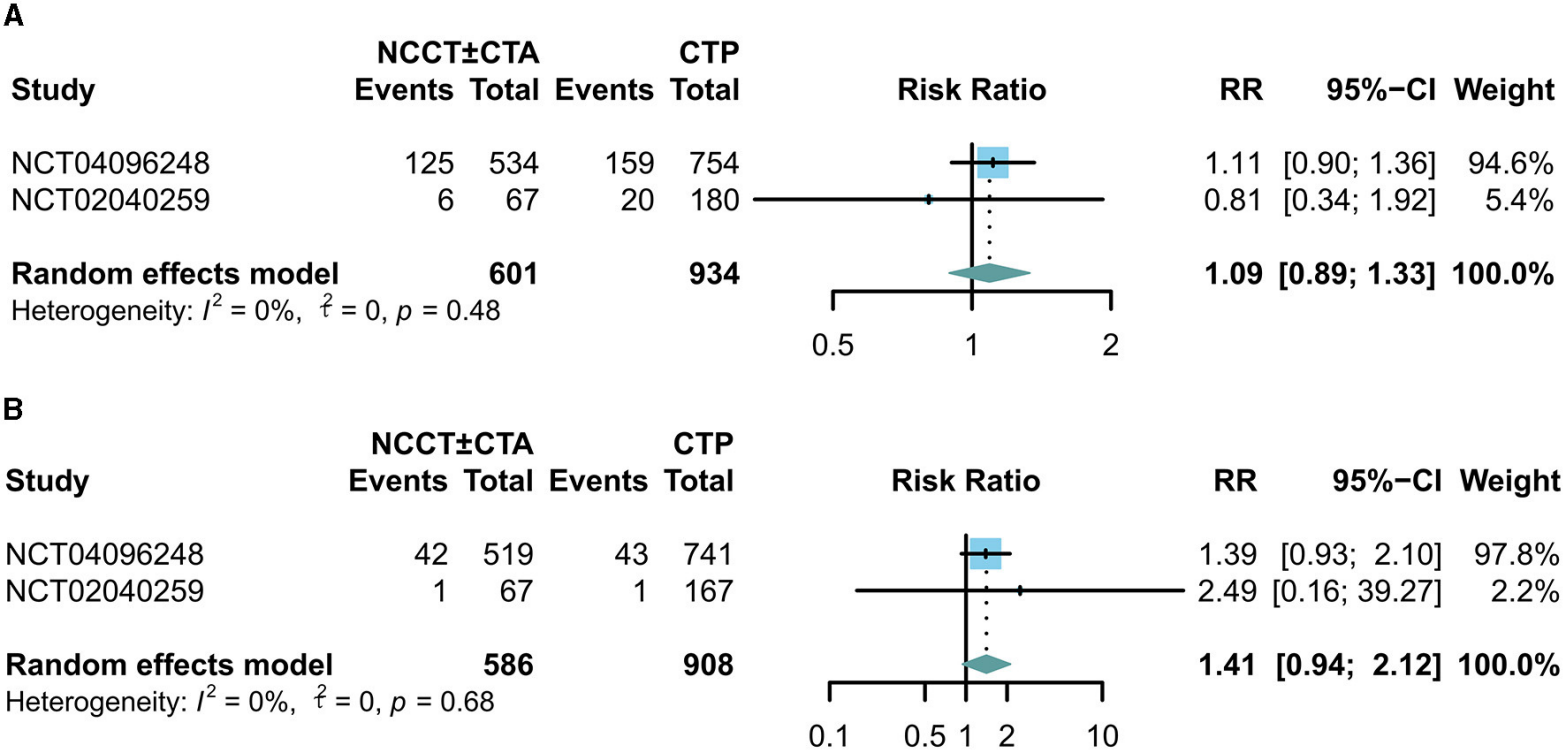
Forest plots for the incidence of adverse events. The diamond indicates the risk ratio (95% confidence interval) for all patients combined. **(A)** 90-day mortality, **(B)** incidence of symptomatic intracranial hemorrhage.

There was no significant difference between the two groups in terms of 90-day mortality (RR: 1.09; 95% CI: 0.89, 1.33; Figure 4A) and the incidence of symptomatic intracranial cerebral hemorrhage (sICH) (RR: 1.41; 95% CI: 0.94, 2.12; Figure 4B).

## 4. Discussion

Our meta-analysis based on two large samples of randomized controlled studies (3, 4) suggests that more basic imaging modalities (NCCT ± CTA) replace advanced imaging to some extent during extended time windows (6, 12). Both studies analyzed the association between imaging modality and the 90-day mRS in an ordered (modified Rankin scale offset) and dichotomous (functionally independent, modified Rankin scale scores 0 ~ 2) manner. To further validate our point, it was discussed whether a more inclusive selection paradigm could be used to allow a larger proportion of patients with extended time windows to be treated and whether they could still maintain significant benefits.

The p imaging paradigm usually relies on the use of NCCT and CTA to simply predict ischemic core volume (9). In contrast, perfusion imaging provides a mismatched ratio to estimate the proportion of salvageable tissue for patient selection (13). Results from two landmark trials show that CTP is extremely beneficial for stroke patients undergoing endovascular treatment options within an extended time window. Nevertheless, the use of perfusion imaging is becoming increasingly controversial. In the SWIFT PRIME trial (10), perfusion imaging did not improve treatment efficacy and was associated with a potential time delay. Similarly, several other trials have shown no significant interaction between CTP mismatch and treatment effectiveness and no association with functional prognosis (14–16).

The first consideration is that acute CTP or MRI is not easily performed in many stroke centers around the world (6, 17). Advanced imaging resources are not readily available, and routine utilization of perfusion imaging with an extended time window does not correspond to reality (17). For the majority of patients who are not eligible for inclusion in the DAWN and DEFUSE3 trials, perfusion imaging is not performed. Moreover, from the standpoint of smaller hospitals, the full implementation of guideline recommendations for CTP/MRI in patients with suspected large-vessel occlusion is more problematic. It represents a triple challenge of technical, logistical, and economic conditions (18). Furthermore, accurate quantification of infarct tissue has been confirmed to have an impact on clinical outcomes in patients with acute ischemic stroke (19); it allows clinicians to determine the precise area affected by the occlusion and the embolic location (20). Currently, various automated CTP software such as RAPID, MIStar, F-STROKE, and Syngo.via, Spher, and Vitrea vary in their measurements of ischemic core volume and semi-dark zone volume, with overestimation or underestimation of infarct core occurring in each (21, 22). Underestimation of the infarct tends to allow the patient to be included in endovascular treatment to restore lost neurological function but may increase the potential risk of reperfusion bleeding (23). In contrast, overestimating the final infarct core and selecting patients for reperfusion treatment based on the concept of CTP mismatch may exclude patients who may benefit from reperfusion (13, 24). Moreover, there remained discrepancies in the time-consuming image processing by various software, which may delay the time for patients to receive treatment (21, 22). A recent study showed that in up to 25% of cases, CTP may not detect an ischemic core at all, especially in isolated deep middle cerebral artery strokes (25).

NCCT has become the first-line imaging method for acute stroke due to its wide applicability, short examination time, and low examination cost (9). NCCT ASPECTS is an easily accessible imaging metric to assess LVO in AIS (26). Although it may be difficult to detect early ischemic changes after stroke with a non-contrast CT, the sensitivity of non-contrast CT increases over time, and its predictive accuracy for irreversible injury may be higher than relative cerebral blood flow (9, 27). At cerebral blood flow <30%, CTP tends to depict larger infarct core volumes compared to NCCT and may underestimate the volume of potentially salvageable brain tissue, whereas NCCT ASPECTS is superior to CTP in correlating with the ischemic core at this time (28, 29). This means that one cannot rely too much on CTP imaging criteria alone for patient selection. Studies have shown that NCCT ASPECTS correlates with CTP core volume in delayed time windows (30). Combining NCCT ASPECTS with single- or multiphase CTA collateral scores can more accurately predict target zone mismatch (26). At the same time, ASPECTS scoring emphasizes the concern for population variability (31). In a recent large multicenter phase III trial, endovascular treatment selection for LVO stroke patients based on the presence or absence of CTA collateral flow was found to be effective and safe (32). Moreover, prospective studies of CTA-based artificial intelligence (AI) software for the detection of LVO stroke patients have also yielded favorable results (33). Of a total of 1,822 CTAs performed, 190 occlusions were identified, of which 142 were in the internal carotid artery terminal (ICA-T) and middle cerebral artery M1 and M2 sites. The detection rates of ICA-T, M1, and M2 occlusions were 100, 93, and 49%, respectively (34). With the continuous optimization of the algorithm, it is believed that clinicians can reduce the number of potentially salvageable patients missed by using AI as an auxiliary tool. The above evidence suggests that relying solely on NCCT ± CTA for endovascular treatment options for LVO patients is feasible and convenient for small stroke centers and, to some extent, superior to CTP (35).

The retrospective nature of most of the studies and some of the limitations associated with small sample sizes, in addition to the substantial heterogeneity of the reported data, require caution in interpreting our findings. Notably, the MR CLEAN LATE (Endovascular treatment of acute ischemic stroke in the Netherlands for late arrivals) and the RESILIENT-Extended (Randomization of Endovascular Treatment in Acute Ischemic Stroke in the Extended Time Window) trials are underway to provide more definitive evidence of simplified imaging protocols in extended time windows (36). In addition, as the algorithm has evolved, NCCT-based machine-learning models have been developed (37). In a recent study, an algorithm called Methinks was able to detect LVO from NCCT alone with reasonable accuracy (38). Meanwhile, another new technique, dCTA-perfusion, uses the existing ultra-fast three-phase trCTA acquisition to extract perfusion information and derive perfusion maps (39). Preliminary evidence suggests that the perfusion metrics obtained are comparable to those of CTP. The rapid results obtained with these new techniques help speed up treatment decisions compared with CTP evaluation. In addition, shorter scan times and fewer image acquisitions reduce radiation exposure (40). Most importantly, the low resource requirements allow for widespread use, benefiting stroke patients. In summary, the larger concern for healthcare practitioners is that whichever imaging selection paradigm is chosen, the goal is not to determine the maximum treatment benefit for the patient but to distinguish the population most likely to benefit from treatment.

## 5. Conclusion

These findings suggest that preoperative imaging evaluation of patients undergoing mechanical thrombectomy for anterior circulation large-vessel occlusion at 6–24 h after onset does not differ significantly from the outcome of patients choosing the NCCT ± CTA modality compared with the CTP modality and significantly reduces the time from onset to arterial puncture. Our findings support the use of extended time window stroke imaging paradigms.

## Data availability statement

The original contributions presented in the study are included in the article/Supplementary material, further inquiries can be directed to the corresponding authors.

## Author contributions

ZZ and FG were the principal investigators, contributed to the writing of the article, designed the study, and developed the analysis plan. FG analyzed the data and prepared the plots. YJ and YZ revised the manuscript and polished the language. YG and ZW supervised the project. All authors have read and approved the final version of the paper.
