# Supplementary material for: Thrombectomy with and without computed tomography perfusion imaging for large-vessel occlusion stroke in the extended time window: a meta-analysis of randomized clinical trials
Source: Front Neurol. 2023 Aug 17;14:1185554. doi: 10.3389/fneur.2023.1185554 (PMC10470654; doi:10.3389/fneur.2023.1185554)
Supplement: Supplementary file 1 [file Data_Sheet_1.docx]

Supplementary Material

**Preoperative imaging selection for large-vessel occlusion stroke at extended time window: a meta-analysis of randomized clinical trials**

**Zheng Zhan^1, †^, Feng Gu^1, †^, Yi Ji^1^, Yu Zhang^1^, Yi Ge^2, *^, Zhong Wang^1, *^**

*** Correspondence:**

Corresponding Author: Ge Yi, Department of Neurology, The Affiliated Changzhou No.2 People's Hospital of Nanjing Medical University, 68# Middle Gehu Road, Changzhou, 213164, Jiangsu Province, People's Republic of China.

Zhong Wang, The First Affiliated Hospital of Soochow University, 188 Shizi Street, Suzhou, Jiangsu Province, 215006, China.

E-mail address: [geyi016@foxmail.com](mailto:geyi016@foxmail.com) or [wangzhong761@163.com](mailto:wangzhong761@163.com)

# Supplementary Figures

The results of the analysis of 90-day ordinal mRS shift between the NCCT±CTA group and CTP group. The diamond indicates the odds ratio (95% confidence interval) for all patients together. From the top to the bottom is (a) CTP, (b)ASPECTS score, (c)age, (d)diabetes, (e)NIHSS score and (f) time last seen well to puncture.


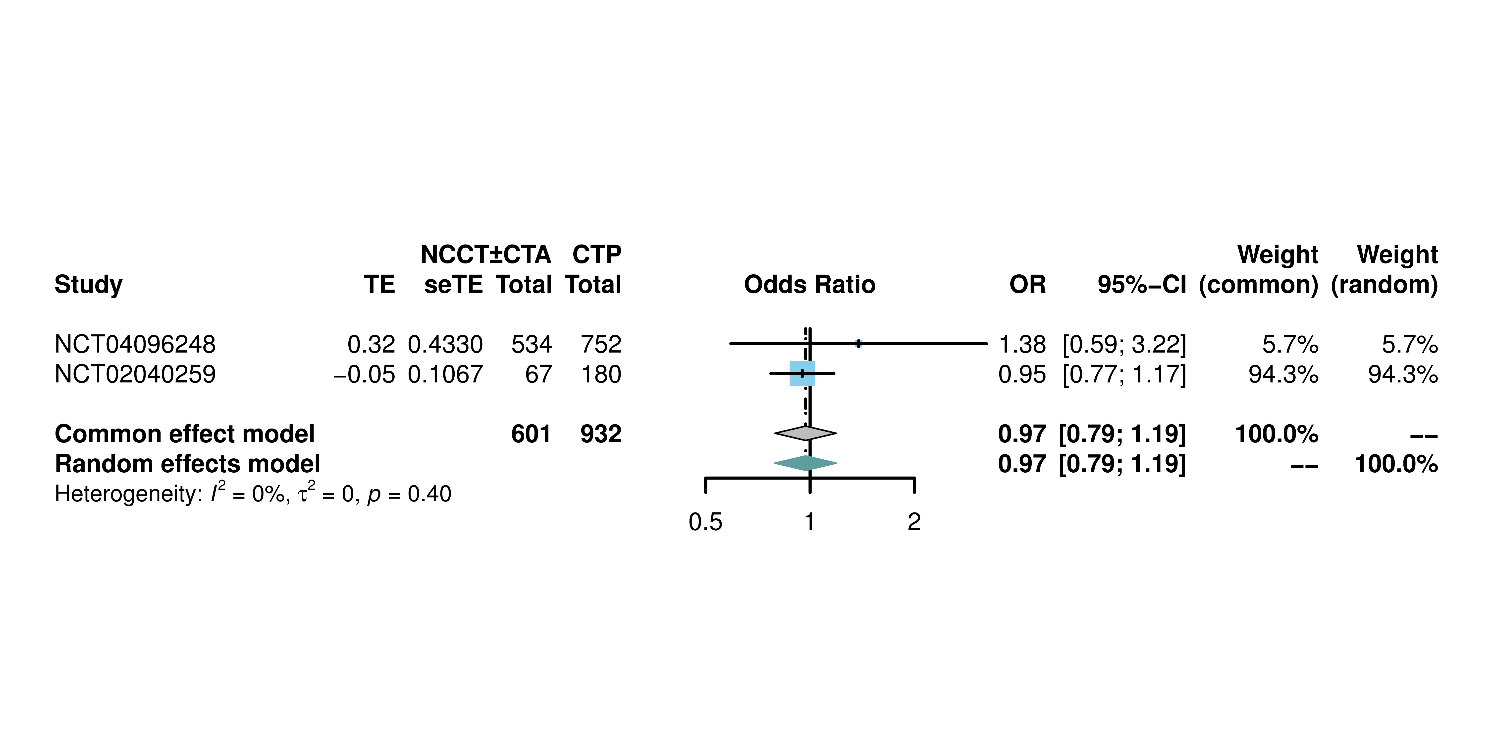


(a)


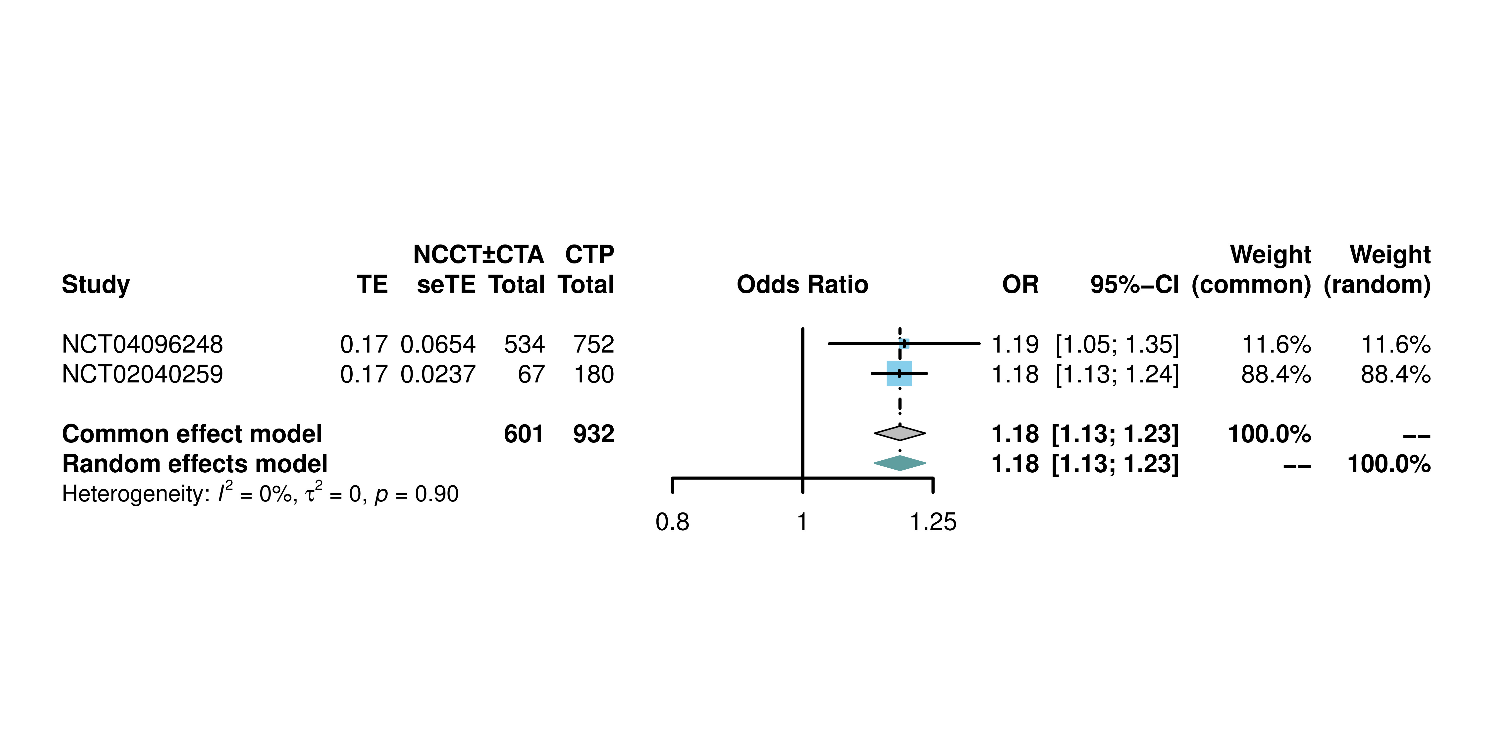


(b)


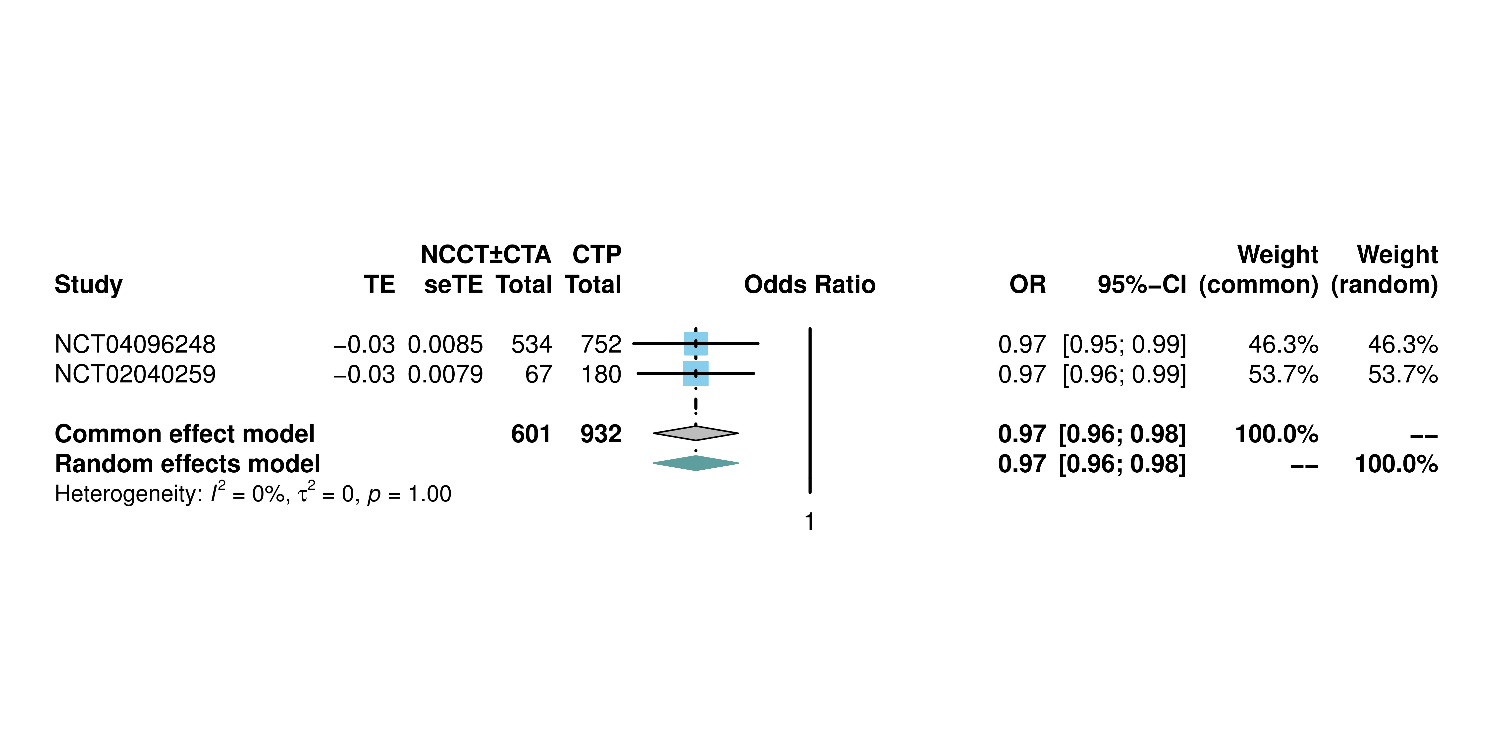


(c)


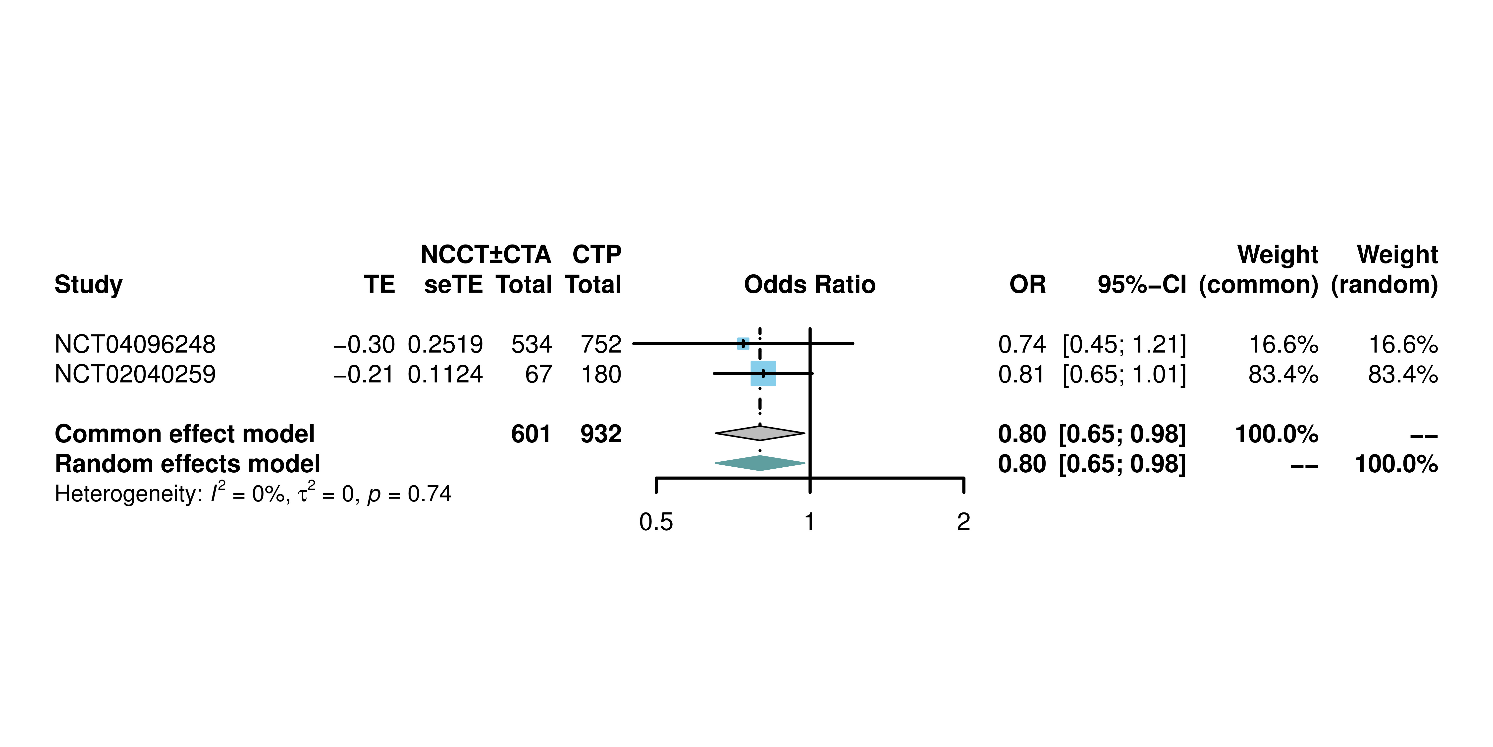


(d)


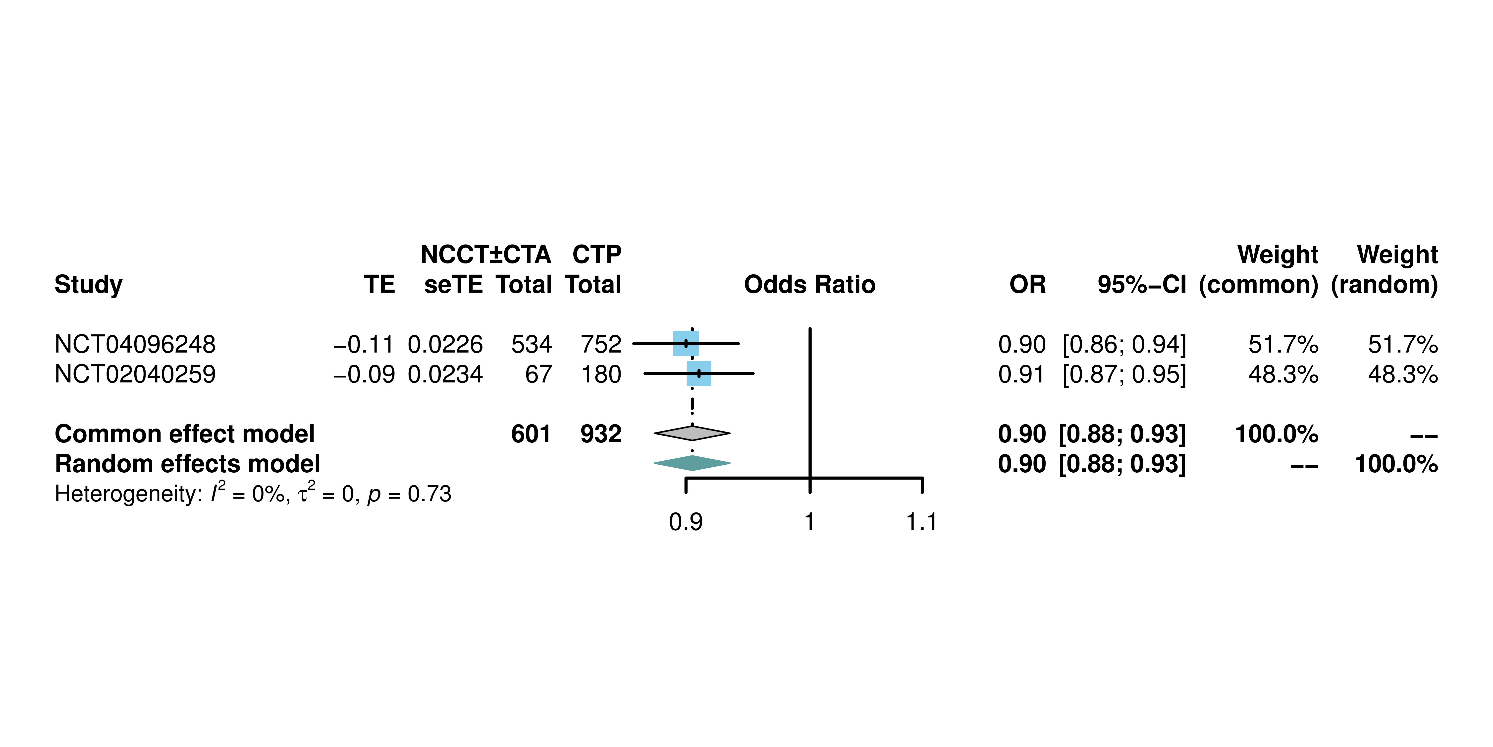


(e)


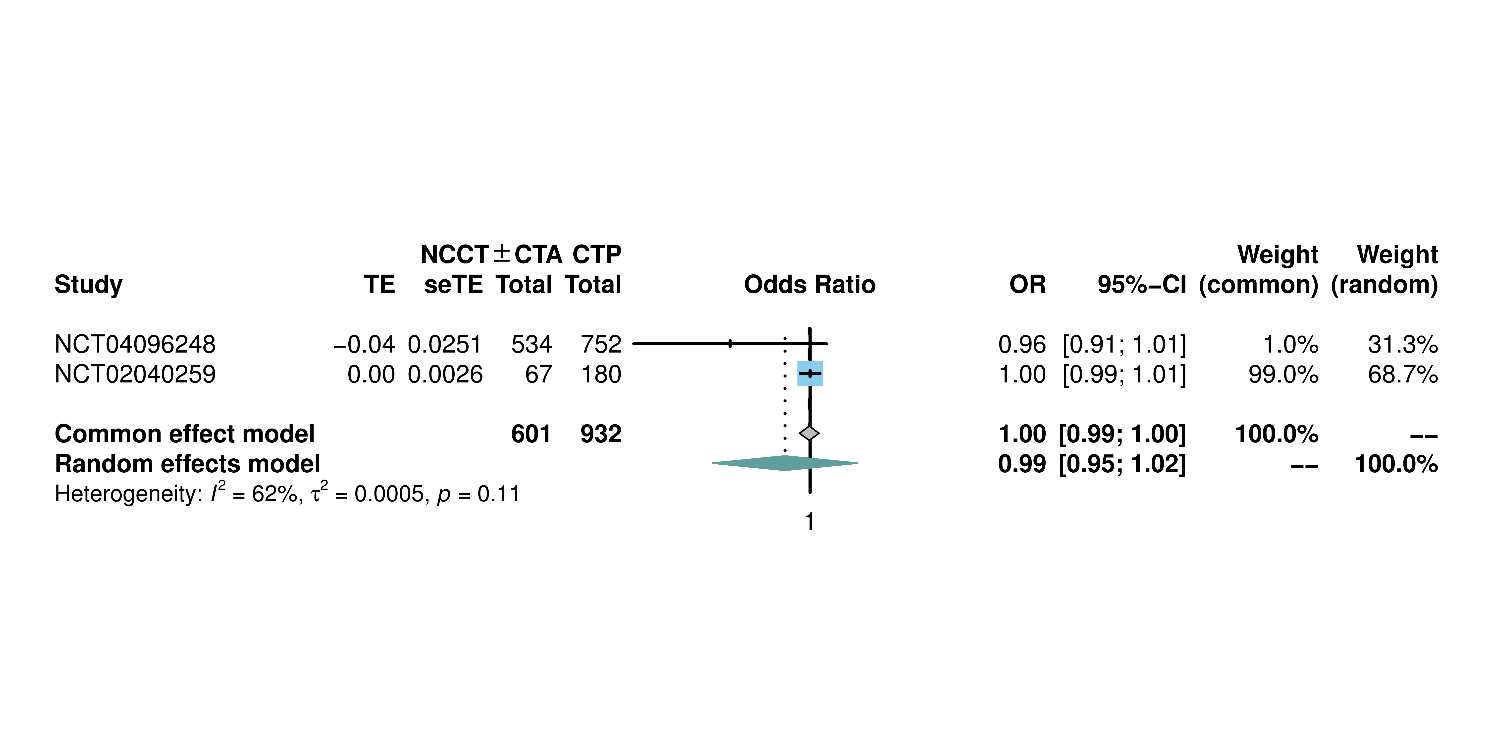


(f)
